# ASL-BIDS, the brain imaging data structure extension for arterial spin labeling

**DOI:** 10.1038/s41597-022-01615-9

**Published:** 2022-09-06

**Authors:** Patricia Clement, Marco Castellaro, Thomas W. Okell, David L. Thomas, Pieter Vandemaele, Sara Elgayar, Aaron Oliver-Taylor, Thomas Kirk, Joseph G. Woods, Sjoerd B. Vos, Joost P. A. Kuijer, Eric Achten, Matthias J. P. van Osch, BIDS maintainers, Stefan Appelhoff, Ross Blair, Franklin Feingold, Rémi Gau, Christopher J. Markiewicz, Taylor Salo, John A. Detre, Hanzhang Lu, David C. Alsop, Michael A. Chappell, Luis Hernandez-Garcia, Jan Petr, Henk J. M. M. Mutsaerts

**Affiliations:** 1 Department of Diagnostic Sciences, Ghent University, Ghent, Belgium; 2 Department of Information Engineering, University of Padova, Padova, Italy; 3 Wellcome Centre for Integrative Neuroimaging, FMRIB Division, Nuffield Department of Clinical Neurosciences, University of Oxford, Oxford, UK; 4 Dementia Research Centre, UCL Queen Square Institute of Neurology, University College London, Queen Square, London, UK; 5 Neuroradiological Academic Unit, Department of Brain Repair and Rehabilitation, UCL Queen Square Institute of Neurology, University College London, Queen Square, London, UK; 6 Faculty of computers and information science, Ain Shams University, Cairo, Egypt; 7 Gold Standard Phantoms, London, UK; 8 Institute of Biomedical Engineering, Department of Engineering Science, University of Oxford, Oxford, UK; 9 Sir Peter Mansfield Imaging Center, School of Medicine, University of Nottingham, Nottingham, UK; 10 Center for Functional Magnetic Resonance Imaging, Department of Radiology, University of California San Diego, La Jolla, California USA; 11 Centre for Medical Image Computing, University College London, London, UK; 12 Department of Radiology and Nuclear Medicine, Amsterdam University Medical Center, Amsterdam Neuroscience, Amsterdam, The Netherlands; 13 C.J. Gorter MRI Center, Department of Radiology, Leiden University Medical Center, Leiden, The Netherlands; 14 Department of Radiology, University of Pennsylvania, Philadelphia, PA USA; 15 Department of Neurology, University of Pennsylvania, Philadelphia, PA USA; 16 Department of Radiology, Johns Hopkins University School of Medicine, Baltimore, Maryland USA; 17 Department of Radiology, Beth Israel Deaconess Medical Center, Harvard Medical School, Boston, Massachusetts USA; 18 Radiological Sciences, Mental Health and Clinical Neurosciences, School of Medicine, University of Nottingham, Nottingham, UK; 19 Nottingham Biomedical Research Centre, Queens Medical Centre, University of Nottingham, Nottingham, UK; 20 Functional MRI Laboratory, University of Michigan, Ann Arbor, Michigan USA; 21 Helmholtz-Zentrum Dresden-Rossendorf, Institute of Radiopharmaceutical Cancer Research, Dresden, Germany; 22 Center for Adaptive Rationality, Max Planck Institute for Human Development, Lentzeallee 94, Berlin, Berlin, 14195 Germany; 23 Department of Psychology, Stanford University, 450 Jane Stanford Way, Stanford, CA 94305 USA; 24 Institute of psychology, Université Catholique de Louvain, Louvain la neuve, MD Belgium; 25 Department of Psychiatry, Perelman School of Medicine, University of Pennsylvania, 3451 Walnut Street, Philadelphia, PA 19104 USA

**Keywords:** Lab life, Brain imaging, Research data

## Abstract

Arterial spin labeling (ASL) is a non-invasive MRI technique that allows for quantitative measurement of cerebral perfusion. Incomplete or inaccurate reporting of acquisition parameters complicates quantification, analysis, and sharing of ASL data, particularly for studies across multiple sites, platforms, and ASL methods. There is a strong need for standardization of ASL data storage, including acquisition metadata. Recently, ASL-BIDS, the BIDS extension for ASL, was developed and released in BIDS 1.5.0. This manuscript provides an overview of the development and design choices of this first ASL-BIDS extension, which is mainly aimed at clinical ASL applications. Discussed are the structure of the ASL data, focussing on storage order of the ASL time series and implementation of calibration approaches, unit scaling, ASL-related BIDS fields, and storage of the labeling plane information. Additionally, an overview of ASL-BIDS compatible conversion and ASL analysis software and ASL example datasets in BIDS format is provided. We anticipate that large-scale adoption of ASL-BIDS will improve the reproducibility of ASL research.

## Introduction

Arterial spin labeling (ASL) is a non-invasive MRI technique for the quantitative measurement of cerebral blood flow (CBF). Important advances in labeling strategies and readout techniques have resulted in sufficient quality for research and clinical use of ASL in the last decade. The 2015 ISMRM Perfusion Study Group ASL recommendations^1^ have led to the implementation of 3D pseudo-continuous ASL (PCASL) as product sequences by GE, Philips, and Siemens, which has improved the comparison of ASL data between multiple centers and studies. Multi-vendor reproducibility studies have shown that ASL CBF values are generally comparable across MRI scanner platforms^2^, particularly when the same labeling scheme is used^3^. Still, acquisition parameters and data management approaches vary across ASL implementations. As accurate ASL image processing and quantification depend on the exact knowledge of the acquisition parameters^1,4^, harmonization and standardization of data structure is potentially a major step forward in guaranteeing comparable CBF quantification, which allows pooling of ASL data.

Comparability of datasets and data sharing is often cumbersome due to inconsistent data management procedures and formats. MR imaging data are heterogeneously organized; each MRI vendor, researcher, and sometimes even each study uses their own ad-hoc structure, which may only contain subsets of the parameters required for image processing. This heterogeneity in data organization, description, and storage complicates the combination of ASL data sets from different sites in multi-center studies, requires additional efforts such as manual input of metadata to perform secondary analyses, and complicates automatic data validation, quality control, image processing and analysis. Unlike contrast-enhanced perfusion MRI, the Digital Imaging and Communications in Medicine (DICOM) standard^5^ is usually not used as the format for processed ASL data. While some ASL-specific DICOM fields are defined, they are incomplete, not mandatory, and rarely used by the vendors^6,7^. Instead, most investigators analyze ASL data in NIfTI^8–10^ format.

The Brain Imaging Data Structure (BIDS), proposed in 2016, is a data storage standard, meeting the need for a structured manner to organize imaging data (https://bids.neuroimaging.io)^11^, which offers a suitable structure to standardize ASL data. The initial BIDS proposal covered anatomical, functional, and diffusion MRI^11^. Subsequently, extensions for magnetoencephalography (MEG)^12^, electroencephalography (EEG)^13^, intracranial EEG (iEEG)^14^, and Positron Emission Tomography^15^ have been incorporated, and several other extension proposals are in development.

In this manuscript, an overview of the development and design choices of the first release of ASL-BIDS is provided. For this first release, only ASL approaches described in the 2015 ASL consensus paper are included^1^. For the remainder of this manuscript, the reader is assumed to be familiar with ASL terminology, which is detailed in the 2015 ISMRM Perfusion Study Group ASL recommendations^1^.

## ASL-BIDS Specification

### ASL approaches included in this first release

The existing efforts for standardization - National Electrical Manufacturers Association (NEMA) DICOM C.8.13.5.14 MR Arterial Spin Labeling Macro^6,7^ and the 2015 ASL consensus paper^1^ - were used as the basis for ASL-BIDS. To facilitate adoption, this extension only supports ASL approaches that were recommended or discussed in the 2015 ASL consensus paper: pulsed (PASL) and (pseudo)-continuous ((P)CASL)^1^. ASL sequence types with single and multiple post-labeling delays (PLDs), for which BIDS could be extended with minimal changes, were also included. Examples include the addition of a single BIDS field to indicate a Look-Locker readout or allowing a scalar value in an array format to support the multiple contrast types of Quantitative STAR labeling of arterial regions (QUASAR)^16,17^.

### ASL-BIDS structure

While ASL can provide information relating to functional activation of the brain, it is most commonly used to measure a fundamental physiological parameter (perfusion) that reflects baseline metabolic demand rather than transient neural activity, distinguishing it from the existing BIDS data type ‘func’. Therefore, a new perfusion data type ‘perf’ for ASL-related data was defined, which can also be used for other perfusion-related BIDS extensions in the future, such as dynamic susceptibility contrast (DSC) MRI^18^.

A consensus was reached to store image volumes in the same order as they were acquired. This preserves data integrity and allows easy review of any temporal effects or artifacts, such as head motion, functional ASL, or reactivity measurements based on, for example, CO2 inhalation or acetazolamide infusion^4^. Additionally, it provides more flexibility for various multi-PLD acquisitions and/or labeling approaches, such as Look-Locker and QUASAR^16,17^. It should be noted, however, that the order in which the DICOM images are exported from the scanner may differ from the acquisition order. In this case, the volumes should be sorted back to the acquisition order when storing in BIDS-ASL format. For similar reasons, a consensus was reached to keep any calibration (“M0”) acquisition coherent with the original acquisition, allowing the ‘m0scan’ to be part of the ASL time-series or stored as a separate file. Although recommended by the 2015 ASL consensus statement, there are still many studies in which ASL is acquired without a separate or integrated M0 acquisition. An ‘M0Type’ field was created that specifies if M0 information was acquired, absent, or if a scalar blood M0 is provided. When a blood M0 value is estimated using a different technique, it is recommended to specify the origin of this estimate in the dataset README file. This includes information on the methodology of the measurement, tissue type where the M0 measurements was performed, and how this was converted to blood M0.

Since ASL relies on fast readout techniques, it is often sensitive to distortion or blurring resulting from B0 inhomogeneities. This distortion can be corrected, for example, using an estimate of a B0 fieldmap. Most existing fieldmap approaches in BIDS could already be applied to ASL, except for the technique that uses two acquisitions with normal and reversed phase-encoding polarity (PEPolar). The ASL-BIDS extension adds the option to store an additional ‘m0scan’ with reversed PEPolar in the ‘fmap’ directory, linked by a field ‘IntendedFor’ to the main ‘m0scan’ with normal PEPolar and following the original BIDS specification for fieldmap images.

### Unit scaling

Floating-point raw MRI images are often scaled during the export to 12-bit or 16-bit DICOM files. Scale factors may differ between the ASL raw time series and the M0 image even for M0 scans integrated into the time series, especially when the mean ASL and M0 signal differs significantly due to the use of background suppression^1^. Proper restoration of the acquired ASL values is crucial for the absolute quantification of perfusion images.

In addition to the standard DICOM scale slope tags, scale factors are either stored in private DICOM fields, specified in a separate sequence parameter file, or need to be requested from the vendor. Traditionally, these scaling factors were applied in the quantification phase of an image processing pipeline. The storage of scaling factors in BIDS would make it unclear if and at which stage these factors had been applied. Furthermore, any scaling factors in private fields may be removed during the default anonymization of DICOM files. Therefore, there are no BIDS fields to describe any M0 or ASL scaling, and all scaling defined in the DICOM file (or other source file type) are expected to be applied to the data during conversion to BIDS. This avoids propagating the heterogeneity of (DICOM) scale factors to BIDS. More vendor and sequence-specific details are provided here: https://bids-standard.github.io/bids-starter-kit/tutorials/asl.html.

### ASL-BIDS requirements

Several BIDS fields were added during the development phase of ASL-BIDS, serving the needs of both clinical users and advanced sequence developers. To obtain consensus on the requirement level for these parameters, we decided to rank requirement levels based on their necessity for quantification. ‘REQUIRED’ fields comprise parameters that are essential for CBF quantification as defined in the 2015 ASL consensus paper^1^. Parameters that may improve quantification or explain systematic differences between scanners or ASL sequences are labeled as ‘RECOMMENDED’. For example, the ‘AcquisitionVoxelSize’ is ‘recommended’ as it can be important to consider for gray matter (GM) mask definition or partial volume correction when the reconstruction resolution is not equal to the acquisition resolution^1^. Other parameters were categorized as ‘OPTIONAL’, although they can still be recommended in specific cases. For example, certain populations with pathology-dependent labeling efficiency may benefit from calibration by phase-contrast flow quantification^19^. The value can then be provided in the field ‘LabelingEfficiency’ with additional details on the estimation methodology given in the dataset README file.

Several non-required pre-existing BIDS fields were defined as required for ASL. Examples include ‘MagneticFieldStrength’, which is required to select default values for blood/tissue T1, T2, and T2*^1,4^. ‘SliceTiming’ lists the times that specify the acquisition time of each slice with respect to the start of the volume acquisition. This is required for calculating the effective post-labeling delay for 2D multi-slice sequences. The ‘RepetitionTimePreparation’ is required for the ‘m0scan’ to compensate for incomplete T1 relaxation^1,4,20^. Additionally, ‘EchoTime’ and ‘FlipAngle’ are required for quantification, especially if they differ between the ASL time series and the ‘m0scan’. Also, the BIDS field ‘TotalAcquiredPairs’, which specifies the exact number of ‘control’-’label’ pairs, is required. This field allows estimating some properties of the sequence, such as SNR, that are otherwise lost when only an average image is exported.

### Specification of the labeling plane

Abnormal quantitative perfusion values can result from suboptimal positioning of the labeling slab. Specific fields describing the position and orientation of the labeling plane are available both in the NEMA DICOM C.8.13.5.14 MR Arterial Spin Labeling Macro^6,7^ and in ASL-BIDS. While these provide a complete description of the exact position with respect to the field-of-view, they do not reflect the placement with respect to vascular anatomy when a manual or semi-automatic approach was employed instead of a fixed position. ASL-BIDS thus provides the option for an additional free-text field ‘LabelingLocationDescription’ and an anonymized screenshot ‘*_asllabeling.jpg’ of the labeling plane planning to better describe the position with respect to the subject’s anatomy.

### ASL-BIDS resources

**Fig. 1:**
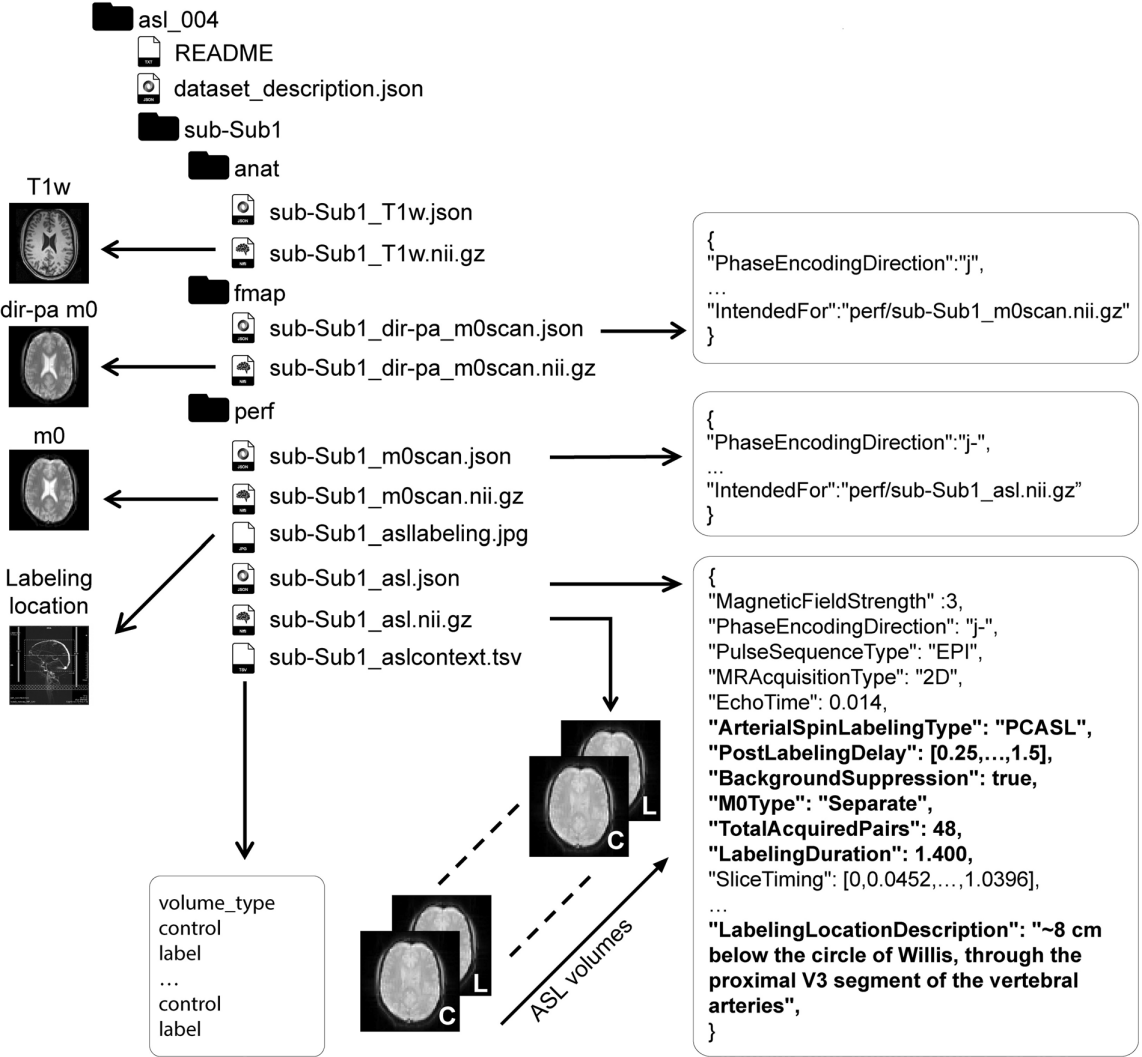
illustrates the ASL-BIDS example dataset asl_004^29^, which is a multi-PLD PCASL dataset composed of several control-label repetitions, and a separate M0 image repeated with an opposite phase-encoding, posterior-anterior (PA), direction for distortion correction. The directory structure, NIfTI files and sidecars such as json and tsv (tab separated values), and other generic files are shown. The ASL data are in the specific folder for perfusion-related files (perf), except for the reversed phase-encoding direction (fmap). For each json, a selection of important fields is shown. Fields given in bold in ASL.json refer to fields that were included manually in DICOM to BIDS conversion as they were not present in the DICOM files.

This BIDS extension for ASL has been implemented in the BIDS validator, which can be used to test BIDS compatibility. Several DICOM to BIDS conversion tools and ASL processing software packages have adopted ASL-BIDS (Tables 1 and 2). In order to provide examples of BIDS compliant ASL datasets, five publicly-available ASL datasets in BIDS format are freely accessible at https://github.com/bids-standard/bids-examples (Table 3), and an illustrative example of one dataset is in Fig. 1. Additionally, the Open Source Initiative for Perfusion Imaging (OSIPI) ASL MRI challenge datasets^21^ are released in BIDS format. Important considerations and exceptions for conversion DICOM to ASL-BIDS, and further explanation of ASL-BIDS fields, are provided in the ASL-BIDS Starter-kit tutorial: https://bids-standard.github.io/bids-starter-kit/tutorials/asl.html.

**Table 1:** Overview of BIDS conversion and valitools compliant with ASL-BIDS.

Tool/Software	Description	Available at
**BIDS-validator**		
*BIDS-validator^11,28^	Validator provided by the BIDS standard, evaluating the compliance of the BIDS-converted dataset with the standard, including metadata and conflicts between data reported in the JSON-file, compared to the data recorded in the NIfTI header.	https://github.com/bids-standard/bids-validator
**DICOM to BIDS conversion tools**		
BIDScoin	A user-friendly, open-source python toolbox. Raw images can easily be converted to BIDS compliant datasets using the Graphical User Interface. Supports ASL-BIDS 1.6.0, uses dcm2niix for conversion.	https://bidscoin.readthedocs.io/en/stable/
dcm2bids	A community-centered project providing a tool for effortless conversion of DICOM images to BIDS format. Uses dcm2niix for conversion.	https://pypi.org/project/dcm2bids/
dcm2niix^24^	A tool for the conversion of images from the DICOM format to the NIfTI format.	https://github.com/rordenlab/dcm2niix
*ExploreASL^4^	A tool for DICOM to ASL-BIDS conversion is included in the ExploreASL processing pipeline. Supports ASL-BIDS 1.6.0, uses dcm2niix for conversion, several further ASL fields are extracted from DICOM directly.	https://github.com/ExploreASL/ExploreASL
heudiconv	A flexible DICOM converter, organizing imaging data into structured directories. Heudiconv provides assistance for the conversion into BIDS format. ASL-BIDS implementation in progress. Uses dcm2niix for conversion.	https://heudiconv.readthedocs.io/en/latest/
pyBIDSconv	A Graphical User Interface tool to convert MRI DICOMs into BIDS format. ASL-BIDS implementation in progress. Uses dcm2niix for conversion.	https://github.com/DrMichaelLindner/pyBIDSconv

An asterisk indicates extensive testing with 51 non-public, clinical datasets from a variety of ASL techniques. All listed tools are free for non-commercial use.

**Table 2:** Overview of software packages compliant with ASL-BIDS. An asterisk indicates extensive testing with 51 non-public, clinical datasets from a variety of ASL techniques.

Tool/Software	Description	Available at
**ASL processing software packages**		
ASLDRO^30^	Open source tool to generate BIDS-compliant simulated ASL digital reference object data. Raw ASL time series comprising control, label and M0 volumes are synthesized from ground truth images according to configurable acquisition and labelling parameters.	https://github.com/gold-standard-phantoms/asldro
ASL-MRICloud^31^	Cloud based tool for processing ASL data. It supports processing of single and multi-PLD data and NIfTI input. JSON and ASL-BIDS are currently not supported.	https://braingps.mricloud.org
ASLPrep^32^	ASL data preprocessing and cerebral blood flow computation pipeline, designed for easy accessibility, state-of-the art interface, and robustness to acquisition variations. BIDS and ASL-BIDS are supported.	https://aslprep.readthedocs.io/en/latest/index.html
ASLtoolbox^33,34^	One of the first Matlab toolboxes for processing ASL data. It contains a graphical user interface and processes single and multi-PLD data. It accepts input in NIfTi, but does not support JSON or ASL-BIDS.	https://cfn.upenn.edu/zewang/ASLtbx.php
BASIL^35^	Toolbox within the FMRIB Software Library providing the tools to analyze ASL datasets with quantification based on Bayesian inference principles. The toolbox accepts both single- and multi-PLD, and also Time-encoded and vessel-selective ASL data in NIfTI-format. Support for ASL-BIDS is in development.	https://asl-docs.readthedocs.io/en/latest/
*ExploreASL^4^	ExploreASL an SPM-based toolbox for processing, statistical analysis, and quality control of ASL datasets. ExploreASL fully supports ASL-BIDS input.	exploreasl.org; https://github.com/ExploreASL/ExploreASL

All listed tools are free for non-commercial use.

**Table 3:** Overview of five freely accessible zeroed-out ASL datasets in BIDS format, available for download at https://github.com/bids-standard/bids-examples; full version on https://osf.io/yru2q/^.29^

Name	Dataset description
asl001	One volunteer scanned on a GE MR750 3 T, using the GE product sequence: a single-PLD PCASL sequence with segmented stack-of-spirals 3D readout and four background suppression pulses. ASL time series consists only of the volumes ‘deltam’ and ‘m0scan’.
asl002	One volunteer scanned on a Philips Achieva 3 T, using the Philips WIP sequence: a single-PLD PCASL sequence with single-shot 2D-EPI and two background suppression pulses, ‘m0scan’ acquired separately.
asl003	One volunteer scanned on a Siemens Trio 3 T, using the Siemens C2P (Bremen) sequence: a multi-PLD PASL sequence with a segmented 3D GRASE readout and two background suppression pulses, ‘m0scan’ acquired separately.
asl004^36,37^	One volunteer scanned on a Siemens Prisma 3 T, using a custom multi-PLD PCASL sequence with a 2D-EPI readout, two background suppression pulses and ‘m0scan’ included in the time series. Dataset includes an additional ‘m0scan’ with reversed phase-encoding direction (pe_polar).
asl005	One volunteer scanned on a Siemens Prisma 3 T, using the Siemens WIP sequence: a single-PLD PCASL sequence with segmented 3D GRASE readout and four background suppression pulses, ‘m0scan’ included in the time series.

All datasets contain a 3D T1W structural scan.

## Discussion

This ASL BIDS extension faced two main challenges compared to existing BIDS data types. Firstly, large variability exists between vendors, scanners, and research labs in the implementation, reconstruction, and export of ASL data. Secondly, ASL measures a quantifiable metric. This renders the accurate reporting of scale slopes essential for quantification, reproducibility, and comparability of ASL studies.

The main limitation of this BIDS extension is that only the ASL approaches recommended in the 2015 ASL consensus paper are included^1^. Advanced ASL approaches such as time-encoded, vessel-encoded, velocity-selective, diffusion-weighted ASL, and functional ASL, may be implemented in a future ASL-BIDS release when their usage has expanded and consensus is reached^22,23^. Another important future extension should be the BIDS definition of ASL image processing derivatives. Users of ASL-BIDS should also be aware that it may not always be possible to derive all mandatory and relevant ASL-BIDS parameters from the header of the primary DICOM image. Therefore, it is important to check that all BIDS mandatory and relevant fields are present, for which the BIDS validator can be a helpful tool: https://bids-standard.github.io/bids-validator/. Most tools for DICOM to BIDS conversion use the dcm2niix tool for conversion to NifTI (dcm2niix; https://github.com/rordenlab/dcm2niix/releases)^24^. While dcm2niix has built-in support for the extraction of some ASL-BIDS fields from DICOM, neither all fields can be currently identified nor are they always present in the DICOM. Furthermore, not all conversion tools support BIDS version 1.6.0, which includes ASL (Table 1). While some processing tools already fully support ASL-BIDS, most ASL pipelines are still working on ASL-BIDS implementation. These pipelines do usually accept the NIfTI format, therefore processing ASL-BIDS data is possible as long as the ASL parameters are manually read from the JSON sidecars and provided to the pipeline (Table 1). Future ASL-BIDS extensions could accommodate new ASL techniques such as velocity-selective ASL, time-encoded ASL, and blood-brain-barrier mapping ASL once their implementation is sufficiently established. Moreover, the extension of BIDS derivatives with ASL-derived images such as CBF images can be important for further standardization. Finally, ASL applications are not limited to the brain^25^. Whereas ASL-BIDS could perhaps be used for other body parts, ASL-BIDS is validated in ASL images of the brain only.

Despite the complexities involved, this effort has already managed to achieve a high level of adoption. Several ASL pipeline developers were included in this effort, leading to the establishment of several major pipelines compatible with ASL-BIDS data so far (Table 3). Additionally, ASL-BIDS was endorsed by the majority of the ASL community (Supplementary Information 1) and is supported by the COST Action CA18206 - Glioma MR Imaging 2.0 (GliMR; http://glimr.eu/)^26^ and the Open Source Initiative for Perfusion Imaging (OSIPI; https://www.osipi.org/). Due to this initial buy-in and the growing recognition of the benefits of standardization^1^, it is expected that ASL-BIDS will have a broad uptake in the community. In the future, ASL-BIDS - in conjunction with OSIPIS’s ASL lexicon^27^ - may encourage NEMA and the MRI vendors to include missing BIDS parameters as DICOM fields.

**Table:** 

**Take-home messages**
1. ASL-BIDS supports the clinically recommended ASL acquisitions described in the 2015 ASL consensus paper. Advanced ASL sequences and ASL-BIDS derivatives may be implemented in a future release. 2. Incompleteness and underuse of NEMA DICOM fields complicate the automation of ASL-BIDS conversion, requiring manual completion of the BIDS fields for ASL. 3. ASL and M0 are stored in their acquisition order to preserve data integrity and allow review of temporal effects. 4. ASL-BIDS conversion should be performed shortly after data acquisition and before any anonymization to guarantee proper scaling.

## Methods

A steering group of ASL experts initiated the ASL-BIDS extension following the principles behind the original BIDS specification^11^. A first draft was prepared, which was shared online from May 2017 until May 2020 with the international ASL community for feedback and suggestions. All comments and suggestions were incorporated or included in a discussion agenda, depending on the impact and clarity of the feedback. This draft was refined during several teleconferences and face-to-face meetings with ASL experts, such as from the European ASL COST-action (BM1103 - Arterial Spin Labelling Initiative in Dementia www.cost.eu/actions/BM1103). In March 2019, the draft was presented at the ISMRM-endorsed International Workshop on Arterial Spin Labeling MRI: Technical Updates and Clinical Experience, held at the University of Michigan. Final concepts and issues were discussed during successive teleconferences within a smaller working group. Discrepancies were resolved by discussion and voting, allowing the finalization of the specification in August 2020.

Simultaneously, five example datasets were made publically available for ASL sequences of GE, Philips, and Siemens. The BIDS-validator^11^ was updated and internally validated using 51 non-public clinical datasets from a variety of ASL acquisition techniques. Additionally, efforts were initiated to adapt existing software analysis tools for ASL-BIDS compatibility^11,28^.

From September 2020 until November 2020, the BIDS extension for ASL, including Appendix XII - Arterial Spin Labeling, and the example datasets and the BIDS validator were disseminated throughout the ASL and BIDS community for testing and endorsement. BIDS version 1.5.0 was released on February 24, 2021, with ASL-BIDS incorporated: https://bids-specification.readthedocs.io/.

## Supplementary information

Supplementary Information 1

## Data Availability

The zeroed-out example datasets developed for this specification are available in the BIDS Examples repository on GitHub, https://github.com/bids-standard/bids-examples; and full version on https://osf.io/yru2q/^29^.

## Code availability

The BIDS validator code is available in the BIDS Validator repository on GitHub, https://github.com/bids-standard/bids-validator.
