# Supplementary material for: ASL-BIDS, the brain imaging data structure extension for arterial spin labeling
Source: Sci Data. 2022 Sep 6;9:543. doi: 10.1038/s41597-022-01615-9 (PMC9448788; doi:10.1038/s41597-022-01615-9)
Supplement: Supplementary file 1 — Supplementary Information 1 [file 41597_2022_1615_MOESM1_ESM.pdf]

Supplementary Information 1

The following investigators endorse ASL-BIDS (sorted on the last name):

Last Name	Full Name	Affiliation(s)
Achten	Rik Achten	Ghent Institute for Functional and Metabolic Imaging (GIfMI), Ghent University, Ghent, Belgium
Aiello	Marco Aiello	IRCCS SDN Foundation, Istituto di Ricerca, Naples, Italy
Amiri	Houshang Amiri	Department of Radiology, Radboud University Nijmegen Medical Centre, Nijmegen, The Netherlands
Anazodo	Udunna Anazodo	Lawson Health Research Institute, London, Ontario, Canada
Asllani	Iris Asllani	Department of Biomedical Engineering, Kate Gleason College of Engineering, Rochester Institute of Technology, Rochester, New York, United States
Baeken	Chris Baeken	Department of Head and Skin, Ghent University Hospital, Ghent, Belgium<br>Ghent Experimental Psychiatry (GHEP) Lab, Ghent University, Ghent, Belgium<br>Department of Psychiatry, free university of Brussels, Brussels, UZ Brussel, Brussels, Belgium<br>Department of Electrical Engineering, Eindhoven University of Technology, Eindhoven, the Netherlands
Bannier	Elise Bannier	Unité visages U746 Inserm-INRIA, IRISA UMR CNRS 6074, University of Rennes, Rennes, France
Barbier	Emmanuel Barbier	Inserm, Grenoble, France
Barker	Gareth J Barker	King's College London, London, United Kingdom
Barkhof	Frederik Barkhof	VU University Medical Center, Amsterdam, The Netherlands
Bastos-Leite	António J. Bastos-Leite	Department of Medical Imaging, Faculty of Medicine, University of Porto, Porto, Portugal
Beaumont	Helen Beaumont	University of Manchester, Manchester, United Kingdom
Bell	Laura Bell	Barrow Neurological Institute, St. Joseph's Hospital and Medical Center, Phoenix, Arizona, United States
Bertoldo	Alessandra Bertoldo	Department of Information Engineering (DEI), University of Padova, Padova, Italy
Beun	Soetkin Beun	Ghent Institute for Functional and Metabolic Imaging (GIfMI), Ghent University, Ghent, Belgium
Bjørnerud	Atle Bjørnerud	The Intervention Centre, Rikshospitalet, Oslo University Hospital, University of Oslo, Oslo, Norway
Bolar	Div Bolar	Division of Neuroradiology, Associate Director for Clinical Translational Imaging Research, Center for Functional Magnetic Resonance Imaging (CFMRI), University of California San Diego, La Jolla, California, United States
Bovo	Stefano Bovo	University of Padova, Padova, Italy
Breutigam	Nora-Josefin Breutigam	Fraunhofer MEVIS Imaging Physics, Bremen, Germany
Caan	Matthan Caan	Department of Biomedical Engineering & Physics, Amsterdam University Medical Center, location AMC, Amsterdam, The Netherlands
Castellaro	Marco Castellaro	Neurology B, Department of Neurosciences, Biomedicine and Movement Sciences, University of Verona, Policlinico G.B. Rossi, P.le L.A. Scuro, 10, 37134, Verona, Italy.

Chappell	Michael Chappell	Wellcome Centre for Integrative Neuroimaging, FMRIB Division, Nuffield Department of Clinical Neurosciences, University of Oxford, United Kingdom<br>Radiological Sciences, Mental Health and Clinical Neurosciences, School of Medicine, University of Nottingham, Nottingham, UK<br>Sir Peter Mansfield Imaging Center, School of Medicine, University of Nottingham, Nottingham, UK<br>Nottingham Biomedical Research Centre, Queens Medical Centre, University of Nottingham, Nottingham, UK
Cavaliere	Carlo Cavaliere	Institute of Research and Medical Care (IRCCS) SDN, Istituto di Ricerca, Naples, Italy
Clement	Patricia Clement	Ghent Institute for Functional and Metabolic Imaging (GIfMI), Ghent University, Ghent, Belgium
Corouge	Isabelle Corouge	Visages U746 Inserm-Inria, IRISA UMR CNRS 6074, University of Rennes 1, Rennes, France
Croal	Paula Croal	University of Oxford, Oxford, United Kingdom
Dai	Weiying Dai	Beth Israel Deaconess Medical Center, Boston, Massachusetts, United States<br>Harvard Medical School, Boston, Massachusetts, United States
De Smet	Stefanie De Smet	Department of Head and Skin, Psychiatry and Medical Psychology, Ghent University Hospital, Ghent University, Ghent, Belgium<br>Ghent Experimental Psychiatry (GHEP) lab, Ghent, Belgium
Detre	John Detre	Brain Science Center, Mahoney Institute for Neuroscience, University of Pennsylvania, Philadelphia, United States
Dijsselhof	Matthijs Dijsselhof	Amsterdam UMC, Amsterdam, the Netherlands
Dolui	Sudipto Dolui	University of Pennsylvania, Philadelphia, United States
Ebrahimi	Tayebeh Ebrahimi	Neuroimaging and Analysis Group, Research Center for Molecular and Cellular Imaging, Tehran, India
Ellmore	Tim Ellmore	The City College of New York, New York, New York, United States
Esteves Padrela	Beatriz Esteves Padrela	Amsterdam UMC, Amsterdam, the Netherlands
Fan	Hongli Fan	Department of Biomedical Engineering, Johns Hopkins University School of Medicine, Baltimore, USA
Ferre	Jean-Christophe Ferre	CHU Rennes, Rennes, France
Figueiredo	Patrícia Figueiredo	Institute for Systems and Robotics, Instituto Superior Técnico, Lisbon, Portugal
Fouto	Ana Fouto	Institute for Systems and Robotics, Instituto Superior Técnico, Lisbon, Portugal
Frisoni	Giovanni Frisoni	Hôpitaux Universitaires de Genève, Genève, Switzerland
Gadmar	Øystein Bech Gadmar	The Intervention Centre, Oslo University Hospital, Oslo, Norway
Gajdoš	Martin Gajdoš	Central European Institute of Technology, Brno, Czechia
Ganji	Sandeep Ganji	Philips Healthcare, Dallas, Texas, United States
Geier	Oliver Geier	Seksjon for diagnostisk fysikk, Oslo Universitetssykehus, Oslo, Norway
Golay	Xavier Golay	Department of Brain Repair and Rehabilitation, Institute of Neurology, University College London, London, United Kingdom
Günther	Matthias Günther	Fraunhofer Institute for Medical Image Computing MEVIS, Bremen, Germany
Guterres	Sofia Guterres	Institute for Systems and Robotics, Instituto Superior Técnico, Lisbon, Portugal

Hales	Patrick Hales	Imaging and Biophysics Unit, UCL Institute of Child Health, London, United Kingdom
Haller	Sven Haller	Affidea CDRC - Radiologie Carouge, Carouge, Switzerland
Henriksen	Otto Henriksen	Copenhagen University Hospital Rigshospitalet, Copenhagen, Denmark
Hernanez-Garcia	Luis Hernandez-Garcia	Functional MRI Laboratory, University of Michigan, Michigan, United States
Ingala	Silvia Ingala	Amsterdam UMC, Amsterdam, the Netherlands
Jahn	Andrew Jahn	Functional MRI Laboratory, University of Michigan, Michigan, United States
Jarutyte	Lina Jarutyte	University of Bristol, Bristol, United Kingdom
Józsa	Tamás Józsa	Department of Engineering Science, Institute of Biomedical Engineering, University of Oxford, Oxford, United Kingdom
Kaczmarcz	Stephan Kaczmarcz	Technische Universität München, München, Germany
Keil	Vera Keil	Amsterdam UMC, Amsterdam, the Netherlands
Kirk	Thomas Kirk	Department of Engineering Science, Institute of Biomedical Engineering, University of Oxford, Oxford, United Kingdom
Knutsson	Linda Knutsson	Medical Radiation Physics, Lund University Hospital, Lund, Sweden
Kuijer	Joost Kuijer	Department of Radiology and Nuclear Medicine, Amsterdam Neuroscience, Amsterdam University Medical Center, Amsterdam, the Netherlands
Leenaerts	Nicolas Leenaerts	KU Leuven, Leuven, Belgium
Lindner	Thomas Lindner	Department of Neuroradiology, University Hospital of Hamburg-Eppendorf, Hamburg, Germany
Liu	Peiying Liu	Division of MR Research, The Russell H. Morgan Department of Radiology & Radiological Science, Johns Hopkins University School of Medicine, Baltimore, Maryland, United States
Lovblad	Karl-Olof Lovblad	HUG, Geneva, Switzerland
Lu	Hanzhang Lu	Department of Biomedical Engineering, Johns Hopkins University School of Medicine, Baltimore, Maryland, United States
MacIntosh	Bradley MacIntosh	Hurvitz Brain Sciences & Physical Sciences, Sunnybrook Research Institute, Toronto, Canada<br>Heart and Stroke Foundation Canadian Partnership for Stroke Recovery, Sunnybrook Research Institute, Toronto, Canada<br>Department of Medical Biophysics, University of Toronto, Toronto, Canada
Majoie	Charles Majoie	Academic Medical Center, Amsterdam, the Netherlands
Maumet	Camille Maumet	IRISA, UMR CNRS 6074, University of Rennes, Rennes, France
Maurel	Pierre Maurel	IRISA, UMR CNRS 6074, University of Rennes, Rennes, France
Mehndiratta	Amit Mehndiratta	Centre for Biomedical Engineering, Indian Institute of Technology, Delhi, India
Moretto	Manuela Moretto	Padova Neuroscience Center, University of Padova, Padova, taly
Morgan	Catherine Morgan	University of Auckland, Auckland, New Zealand
Moyaert	Paulien Moyaert	Ghent University, Ghent, Belgium

Murphy	Kevin Murphy	CUBRIC, Cardiff University, Cardiff, Wales
Mutsaerts	Henk-Jan Mutsaerts	Department of Radiology and Nuclear Medicine, Amsterdam University Medical Center, Amsterdam Neuroscience, Amsterdam, The Netherlands<br>Ghent Institute for Functional and Metabolic Imaging (GIfMI), Ghent University, Ghent, Belgium
Narayana	Ponnada A. Narayana	Department of Diagnostic and Interventional Imaging, UTHealth at Houston, Houston, Texas, United States
Nordhøy	Wibeke Nordhøy	The Intervention Centre, Oslo University Hospital, Oslo, Norway
Noseworthy	Michael Noseworthy	St. Joseph’s Healthcare, Imaging Research Centre (IRC), Toronto, Ontario, Canada
Okell	Thomas Okell	Wellcome Centre for Integrative Neuroimaging, FMRIB Division, Nuffield Department of Clinical Neurosciences, University of Oxford, Oxford, United Kingdom
Özsunar	Yelda Özsunar	Adnan Menderes University, Faculty of Medicine, Department of Radiology, Aydin, Turkey
Ozturk-Isik	Esin Ozturk-Isik	Department of Biomedical Engineering, Yeditepe University, Istanbul, Turkey
Pålhaugen	Lene Pålhaugen	Department of Neurology, Akershus University Hospital, Lørenskog, Norway
Parkes	Laura Parkes	Reader and Lead for Manchester Neuroimaging, University of Manchester, Manchester, United Kingdom
Paschoal	Andre Paschoal	University of Sao Paulo, Sao Paulo, Brazil
Pasternak	Maurice Pasternak	Department of Radiation Oncology, Sunnybrook Health Sciences Centre, Toronto, Canada
Petersen	Esben Petersen	University Medical Center Utrecht, Utrecht, Netherlands
Petr	Jan Petr	Institute of Radiopharmaceutical Cancer Research, Helmholtz-Zentrum Dresden-Rossendorf, Dresden, Germany
Pintor	Nandor Pintor	Dent Neurologic Institute, Amherst, New York, United States
Pinto	Joana Pinto	Institute of Biomedical Engineering, University of Oxford, Oxford, United Kingdom
Pizzini	Francesca Pizzini	University Hospital Verona, Verona, Italy
Preibisch	Christine Preibisch	Technische Universität München, München, Germany
Qin	Qin Qin	FM Kirby Centre, Kennedy Krieger Institute, Johns Hopkins University, Baltimore, Maryland, United States
Reneman	Liesbeth Reneman	Dept. of Radiology and Nuclear Medicine, Amsterdam University Medical Centers, location AMC, Amsterdam, the Netherlands
Rostrup	Egill Rostrup	Functional Imaging Unit, Department of Diagnostics, Glostrup Hospital, Glostrup, Denmark
Rubinski	Anna Rubinski	University of Munich, Munich, Germany
Schmid	Sophie Schmid	Dept. Radiology, Leiden University Medical Center, Leiden, the Netherlands
Schrantee	Anouk Schrantee	Department of Radiology and Nuclear Medicine, Amsterdam Neuroscience, Amsterdam University Medical Center, Amsterdam, the Netherlands
Seara	Maria A. Fernandez Seara	Center for Applied Medical Research, University of Navarra, Pamplona, Spain
Selnes	Per Selnes	University of Oslo, The Intervention Centre, Rikshospitalet, Oslo University Hospital, Oslo, Norway
Sennesael	Nathan Sennesael	Ghent Institute for Functional and Metabolic Imaging, Ghent University, Ghent, Belgium

Siero	Jeroen Siero	Vascular Brain Imaging Group, Universitair Medisch Centrum Utrecht, Utrecht, the Netherlands<br>Spinoza Centre for Neuroimaging Amsterdam, Amsterdam, The Netherlands
Silvestri	Erica Silvestri	Department of Information Engineering, Padova Neuroscience Center, University of Padova, Padova, Italy
Skurdal	Mikjel Skurdal	Department of Radiology, Rikshospitalet, Oslo, Norway
Smits	Marion Smits	Department of Radiology & Nuclear Medicine, Erasmus MC, Rotterdam, The Netherlands
Sokolska	Magdalena Sokolska	UCL Institute of Neurology, Queen Square, London, United Kingdom
Sollmann	Nico Sollmann	Technische Universität München, München, Germany
Sourbron	Steven Sourbron	University of Sheffield, Sheffield, United Kingdom
Stritt	Michael Stritt	Mediri GmbH, Heidelberg, Germany
Sunaert	Stefan Sunaert	Radiology, University Hospitals Leuven, Leuven, Belgium<br>Translational MRI, KU Leuven, Leuven, Belgium
Suzuki	Yuriko Suzuki	Wellcome Centre for Integrative Neuroimaging, FMRIB, University of Oxford, Oxford, United Kingdom
Thomas	Binu Thomas	UT Southwestern Medical Center, Dallas, Texas, United States
Thomas	David Thomas	UCL Institute of Neurology, Queen Square, London, United Kingdom
Vaclavu	Lena Vaclavu	Dept. of Radiology, Leiden University Medical Center, Leiden, the Netherlands
Van Osch	Thijs Van Osch	C.J. Gorter Center for High Field MRI, Department of Radiology, Leiden University Medical Center, Leiden, the Netherlands
van Stralen	Marijn van Stralen	Image Sciences Institute, Center for Image Sciences, University Medical Center Utrecht, Utrecht, the Netherlands
Villani	Umberto Villani	University of Padova, Padova, Italy
Vos	Sjoer Vos	UCL, London, United Kingdom
Vossough	Arastoo Vossough	Children's Hospital of Philadelphia and Hospital of the University of Pennsylvania, Philadelphia, United States
Wang	Danny JJ Wang	Laboratory of FMRI Technology (LOFT), Mark & Mary Stevens Neuroimaging and Informatics Institute, Keck School of Medicine, University of Southern California, California, United States
Wang	Ze Wang	University of Maryland School of Medicine, Baltimore, Maryland, United States
Warnert	Esther Warnert	Department of Radiology & Nuclear Medicine, Erasmus MC, Rotterdam, The Netherlands
Warnking	Jan Warnking	Inserm U836, Grenoble Institut des Neurosciences, Grenoble, France
Wiegers	Evita Wiegers	University Medical Center Utrecht, Utrecht, Netherlands
Willekens	Sanneke Willekens	University of Twente, Twente, the Netherlands
Wink	Alle Meije Wink	Amsterdam UMC, Amsterdam, the Netherlands
Woods	Joseph Woods	CFMRI, Department of Radiology, University of California San Diego, La Jolla, California, United States
Yang	Wang Yang	Center for Neuroimaging, Indiana University School of Medicine, Indianapolis, Indianapolis, United States

Yen	Cecil Chern-Chyi Yen	NIH, Bethesda, Maryland, Unites States
Zhao	Li Zhao	University of Virginia, Charlottesville, Virginia, United States
Zhao	Moss Zhao	Department of Radiology, Stanford, Stanford University, California, United States
